# Pollution and cardiovascular health: A contemporary review of morbidity and implications for planetary health

**DOI:** 10.1016/j.ahjo.2022.100231

**Published:** 2022-12-06

**Authors:** Earl Goldsborough, Medha Gopal, John William McEvoy, Roger S. Blumenthal, Alan P. Jacobsen

**Affiliations:** a Johns Hopkins University School of Medicine, Baltimore, MD, USA; b Saint George's University School of Medicine, University Centre Grenada, West Indies, Grenada; c National Institute for Prevention and Cardiovascular Health, National University of Ireland Galway, Galway, Ireland; d Ciccarone Center for the Prevention of Cardiovascular Disease, Division of Cardiology, Department of Medicine, Johns Hopkins University School of Medicine, Baltimore, MD, USA

**Keywords:** Cardiovascular disease, Pollution, Planetary health, Primary prevention, Preventive cardiology

## Abstract

Pollution is a leading cause of premature morbidity and mortality and an important risk factor for cardiovascular disease. Convincing data predict increased rates of cardiovascular morbidity and mortality with current and projected pollution burden trends. Multiple classes of pollutants – including criteria air pollutants, secondhand smoke, toxic steel pollutants, and manufactured chemical pollutants – are associated with varied cardiovascular disease risk profiles. To reduce the future risk of cardiovascular disease from anthropogenic pollution, mitigation strategies, both at the individual level and population level, must be thoughtfully and intentionally employed. The literature supporting individual level interventions to protect against cardiovascular disease is growing but lacks large clinical trials. Population level interventions are crucial to larger societal change and rely upon policy and governmental support. While these mitigation strategies can play a major role in maintaining the health of individuals, planetary health – the impact on human health because of anthropogenic perturbation of natural ecosystems – must also be acknowledged. Future research is needed to further delineate the planetary health implications of current and projected pollutant burden as well as the mitigation strategies employed to attenuate future pollutant burden.

## 1 Introduction

The body of literature supporting pollution as a major risk factor for cardiovascular disease (CVD) continues to grow [1], [2]. The *Lancet* Commission on pollution and health cites pollution as the leading environmental cause of morbidity and premature mortality [3]. The Global Burden of Diseases study estimated that pollution was responsible for 5.5 million deaths due to CVD in 2019 [4], and epidemiologic studies estimate 6–9 million attributable deaths each year, through 2060, with the current and projected global pollution burden [5]. Despite the recognition of this public health hazard, there is a paucity of data to support actionable strategies to mitigate the effects of pollution on cardiovascular (CV) health.

The deleterious effects of anthropogenic pollution extend beyond human health and are associated with biodiversity loss (eg, species extinctions, decreased species abundances) and perturbations of biodiversity distribution and composition (eg, alterations in genetic diversity and habitat fragmentation) in ecosystems around the world [6], [7]. In 2019, the Intergovernmental Science-Policy Platform on Biodiversity and Ecosystem Services (IPBES) identified pollution as one of the five direct drivers of global biodiversity loss [8]. Biodiversity loss is driven through multiple mechanisms, including acid rain resulting in the acidification of water and soil environments, air pollution burden leading to reproductive failure and teratogenicity in exposed wildlife, and eutrophication – the excessive deposition of nitrogen is aquatic ecosystems – causing fish death and marine disequilibrium [9].

A 2020 study, used a global dataset to analyze the cumulative human footprint, identified over 20,000 terrestrial vertebrate species experiencing intense pressures resulting from anthropogenic threat complexes (ATCs) – the combinations of anthropogenic pressures acting on a natural ecosystem [10]. Current rates of species extinction are estimated to be 1000-fold greater than the presumed background rate of extinction, attributable to anthropogenic influences [11]. Such biodiversity loss has negative impacts on food security, regulation of infectious disease, and the provision of medicine and medical research with associated health effects [12]. Accordingly, the mitigation strategies employed to counteract the health effects of air pollution must incorporate planetary health (i.e., the health impacts of disruption to the earth's natural systems caused by humans). Planetary health describes how anthropogenic perturbations of natural ecosystems cyclically impacts human health [13]. For example, the mass production of persistent plastics has resulted in their permeation into aquatic ecosystems where such microplastics are consumed by fish, which humans then consume [14], [15].

Prior reviews evaluating the relationship between pollution and CV health have focused primarily on the pathophysiology and epidemiological impact of pollution. In this review, evidence buttressing the deleterious effects of air pollution on CV health are evaluated, recommendations for practical strategies to mitigate future pollution-mediated CVD risk are proposed, and the implications on planetary health of these strategies are discussed.

## 2 Cardiovascular morbidity and mortality

### 2.1 Criteria air pollutants

The United States Environmental Protection Agency (EPA) recognizes six major (i.e., criteria) air pollutants; particulate matter with a diameter < 2.5 μm (PM_2.5_), tropospheric ozone, nitrous oxides, carbon monoxide, sulfur dioxide, and lead. Each criterion air pollutant is associated with its own CV risk profile (Table 1) [16]. PM refers to the heterogeneous mixture of solid particles and liquid droplets found in ambient air and is categorized by the maximum average diameter of these particles, with PM_2.5_ having the strongest association with CVD [17]. The World Health Organization (WHO) recommends an air quality guideline (AQG) level for annual PM_2.5_ exposure of <5 μg/m^3^ along with four interim targets levels – with an optimal interim target annual exposure of <10 μg/m^3^ [18]. This AQG level of <5 μg/m^3^ is derived from projections of non-accidental mortality reported in a 2020 systematic review, which projects a linear hazard ratio (HR) of 1.08 per 10 μg/m^3^ increase in PM_2.5_ [19].

**Table 1:** Air pollutants and cardiovascular disease associations.

Air pollutants and cardiovascular disease associations		
Criteria air pollutants	Air pollutants	Associated cardiovascular disease
	Carbon monoxide [23], [41], [42]	• Coronary heart disease • Heart failure • Metabolic syndrome • Stroke • Vascular disease
	Tropospheric ozone [30], [31], [33]	• Cardiopulmonary mortality • Coronary heart disease • Stroke
	Lead [48]	• Coronary heart disease • Stroke
	Nitrous oxide [36], [37], [38]	• Atherosclerosis • Cardiovascular mortality • Heart failure • Hemorrhagic stroke • Hypertension • Myocardial infarction • Stroke
	Particulate matter 2.5 [20], [21], [22], [23], [24], [25], [27], [29]	Short-term: • Atrial fibrillation • Dysautonomia • Heart failure exacerbation • Heart rate variability • Hemorrhagic stroke • Hypertension • Myocardial infarction Long-term: • Acute myocardial infarction • Cardiopulmonary mortality • Ischemic heart disease • Stroke
	Sulfur dioxide [44], [45]	• Arrhythmias • Cardiovascular mortality • Coronary heart disease • Heart failure • Left ventricular dysfunction
Non-criteria Air Pollutants	Climate change [113]	• Myocardial Infarction • Stroke
	Secondhand smoke [49]	• Coronary heart disease
	Toxic steel pollutants [51], [52]	• Coronary heart disease • Stroke
	Manufactured chemical pollutants [54], [55]	• Cardiovascular mortality • Coronary heart disease • Hypertension • Stroke

Short-term (i.e., <24 h) exposure to increased PM_2.5_ is associated with increased risk of dysautonomia, hypertension, atrial fibrillation, decompensated heart failure, MI, and CVD mortality [20], [21], [22], [23], [24], [25]. A 2013 study using exposure models demonstrated a 2.8 % absolute increase in PM-related mortality for every 10 μg/m^3^ increase in short-term PM_2.5_ exposure [26]. Long-term exposure to elevated levels of PM_2.5_ is associated with increased risk of acute MI, ischemic heart disease (IHD), and stroke [27]. A 2016 study associated a 50 ng/m^3^ increase in PM_2.5_ with an 18 % increase in adverse coronary events, IHD, and hypertension [28]. Long-term exposure to elevated levels of PM_2.5_ is also associated with a 6–13 % increased risk of cardiopulmonary mortality for every 10 μg/m^3^ increase in exposure to PM_2.5_ [29].

Ozone is a gas composed of three oxygen atoms and can be classified as stratospheric or tropospheric, based on where it is generated. Tropospheric, or ground-level, ozone is the product of combustion reactions between nitrous oxides and volatile organic compounds emitted from fossil fuel combustion. Tropospheric ozone has important implications on CV health. A 10 μg/m^3^ increase in same day and preceding day tropospheric ozone is associated with a 1.7–1.8 % increased risk of mortality in patients with a previous admission for acute MI [30], [31]. A large prospective study demonstrated that long-term exposure to tropospheric ozone increased the risk for overall pulmonary and circulatory mortality [32]. Additionally, ozone is associated with an increased risk of all-cause mortality, IHD, and stroke – associations which persist nearly unchanged when controlling for PM [33].

Nitrogen oxides are a class of nitrogenous air pollutants formed by high-temperature fossil fuel combustion, overwhelmingly derived from engine combustion reactions in vehicles [34], [35]. Short-term exposures to nitrogen oxides are associated with increased risk of cardiovascular death, MI, and hemorrhagic stroke [36]. Longitudinal studies have established an association between long term nitrogen dioxide exposure and all-cause mortality, atherosclerosis, hypertension, stroke, and heart failure [37], [38].

Carbon monoxide is a gaseous pollutant generally produced by the combustion of organic material, often fossil fuels. Motor vehicle emission is the greatest source of carbon monoxide [39], [40]. Carbon monoxide poisoning is associated with an increased risk of heart failure [23], and increased exposure is associated with both metabolic syndrome and vascular disease [41]. A 2018 study found a 2 % increased risk of mortality from cardiovascular disease, coronary heart disease, and stroke with a 1 mg/m^3^ increase in exposure above average carbon monoxide concentrations from the prior day [42].

Sulfur dioxide is an important gaseous pollutant that is primarily produced as a byproduct of fossil fuel combustion [43]. Short-term exposures to sulfur dioxide are associated with increased risk of IHD [44]. Additional studies have demonstrated that sulfur dioxide promotes left ventricular dysfunction, increased CV mortality and morbidity, arrhythmia, and ischemic heart failure [45]. A longitudinal study recently yielded data suggesting that long-term exposure to sulfur dioxide increases risk of incident coronary heart disease [46].

Lead is a toxic steel pollutant; toxic steel pollutants are metals and metal compounds, which have deleterious effects on human health [47]. Lead is one of the best studied toxic steel pollutants; a 2018 meta-analysis comparing top and bottom thirds of baseline lead levels demonstrated levels of lead consumption resulting in lead levels in the top tercile are associated with a 1.43; 1.85; and 1.63 relative risk of CVD, CHD, and stroke, respectively [48].

### 2.2 Other pollutants

Other notable pollutants include passive, or secondhand, smoke, additional toxic steel pollutants (eg, arsenic, cadmium, and copper), and manufactured chemical pollutants. Passive smoking, or exposure to secondhand smoke, has been associated with an increased risk of CHD [49]. A recent meta-analysis estimates a 28 % increased risk of CVD with secondhand smoke exposure, with mortality highest (adjusted HR = 1.26) in those exposed at home, work, and public spaces [50]. The association between passive smoking and cardiovascular disease is not fully understood and requires further study.

A 2018 meta-analysis comparing top and bottom thirds of baseline toxic steel pollutant (eg, arsenic, cadmium, and copper) levels demonstrated levels of consumption resulting in serum levels in the top tercile are associated with a 1.30; 1.23; and 1.15 relative risk of CVD, CHD, and stroke, respectively for arsenic. Cadmium portends a 1.33; 1.29; and 1.72 relative risk of CVD, CHD, and stroke, respectively. Copper is associated with a 1.81; 2.22; and 1.29 relative risk of CVD, CHD, and stroke, respectively [48]. Arsenic, lead, and cadmium exhibit a linear dose-response relationship with adverse CV outcomes, mediated largely through atherogenesis [51], [52].

Manufactured chemical pollutants are also referred to as persistent organic pollutants given the longevity of their impact, and they refer to the synthetic chemicals largely resistant to environmental degradation, which became commercialized during industrialization following World War II [53]. Common manufactured chemical pollutants with implications on CV health include persistent organic pollutants, plastic exudates (eg, bisphenol A and phthalates), low molecular weight hydrocarbons (LMWHCs), polynuclear aromatic hydrocarbons (PAH), and perfluoroalkyl substances (PFAS). The CV toxicity of these pollutants is related to the total serum lipophilic chemical load and greater exposure is associated with increased risk of overall CV mortality and stroke [54], [55].

### 2.3 Climate change

Air pollution and climate change are primarily caused by fossil fuel combustion [56]. Climate change affects human health through extreme weather events, heat stress, air pollution, infectious diseases, malnutrition, and other factors such as migration and displacement, as outlined by The Lancet Countdown International Collaboration on Health and Climate Change [57]. Multiple factors mediate the association between climate change and cardiovascular events (eg, heat, ambient air pollution, vector-borne disease and psychosocial factors) [58], [59]. For every 1 °C increase in temperature above a baseline of 26 °C, there is an associated 4.3 increase in CVD mortality [60]. Furthermore, increased temperatures are associated with increased mortality related to PM exposure [61]. Climate change mitigation strategies that reduce the rate of greenhouse gases released can be expected to have positive impacts on air pollution levels.

### 2.4 Population level interventions

Population and individual-level interventions are required to address the health impact of pollution. Population-level interventions aim to reduce the generation of, and exposure to, pollutants. They differ from individual-level interventions in that population-level interventions presuppose legislative or civic action to buttress the proposed initiatives– given the breadth of the populations(s) employing these strategies.

The EPA is the principal governmental agency responsible for regulating environmental exposures to pollutants in the US [62]. The EPA has promoted multiple initiatives which have been codified (Table 2), including the *Clean Air*-, *Clean Water*-, *Toxic Substances Control*-, *Resource Conservation and Recovery*-, and the *Comprehensive Environmental Response, Compensation, and Liability Acts,* which regulate the introduction of, and exposure to, toxic pollutants [63]. These initiatives have been effective, but complacency threatens the viability of these improvements [64].

**Table 2:** U.S. EPA acts to address population at the level of the population.

United States environmental protection agency acts to address pollution at the level of the population	
Act	Function
Clean Air Act	Regulates air pollution by requiring federal and state regulations for air emissions from stationary and mobile sources
Clean Water Act	Regulates the discharge of pollutants into the waters and establishes quality regulation standards for surface water
Toxic Substances Control Act	Allows the EPA to impose reporting, record-keeping, testing requirements, and restrictions on chemical substances and mixtures
Resource Conservation and Recovery Act	Provides a framework for managing hazardous and non-hazardous solid waste.
Comprehensive Environmental Response, Compensation, and Liability Act	Responds to abandoned, uncontrolled hazardous waste sites and provides a fund (i.e., superfund) to clean up abandoned hazardous waste sites

Broad classes of future considerations for population-level interventions (Table 3) include concerted legislative efforts to limit industrial emissions, efforts to limit transportation-related emissions, and efforts to mitigate or stop the progression of global warming [2]. Additional considerations include concerted legislative and civic efforts to improve access to safe and accessible recreational areas (eg, community parks) and to increase accessibility to clean and safe housing for citizens with regulations mandating government-funded upkeep of community housing. This would limit the exposure to household pollutants in government housing. Additionally, there is a need for environmental justice for the social determinants of health with respect to the rectification of governmental housing divisions, which disproportionately expose government housing residents to increased levels of PM_2.5_ [65].

**Table 3:** Population-level interventions to mitigate the effects of, and exposure to, pollutants.

Population-level interventions	
Interventions	Effects
Limiting industrial emissions	Promote ecologically sustainable methods of production
Limiting transportation-related emissions	Reduce air pollutant burden in areas of high travel
Regulate climate change	Attenuate effects of global warming
Increase access to recreational areas	Mitigate exposure to indoor pollutants
Environmental justice	Limit accumulation of indoor and ambient pollutant exposure

Predictive models estimate a 4 % reduction in premature mortality in the US due to PM_2.5_ exposure with strict adherence to an exposure standard of 12 μg/m^3^, the original AQI standard established in 2012 [66]. A 2021 study yielded data suggesting that concerted governmental efforts to disseminate filtration air masks are likely to reduce exposure (PM_2.5_ removal efficiency of filtration air masks = 70–81 %); however, endorsement of regular use is needed, as many people who consistently wear air filtration masks outdoors do so for warmth rather than reduced exposure to ambient air pollutants [67].

Government policy to promote population-level strategies to mitigate pollution burden and exposure has been demonstrated to be efficacious outside of the US, with implementation of the Chinese *Air Pollution Prevention and Control Action Plan of 2013* being associated with 6.8 % reduction in annual deaths attributable to PM_2.5_ pollution over the first four years of its implementation [68]. Within the US, the Clean Air Act prevented an estimated 205,000 premature deaths as of 2012 [69] and by 2020, has likely prevented ~230,000 adult deaths due to PM and 200,000 deaths due to AMI [70].

With multiple, concurrent initiatives to reduce pollutant burden and exposure, there needs to be an effort to evaluate the sustainability of these efforts, using this information to guide future efforts. Air pollution accountability, a growing field which assesses the efficacy of regulatory actions, will be important going forward as additional initiatives are conceptualized and effectuated [71].

### 2.5 Individual level interventions

Individual interventions (Table 4) are crucial in mitigating the adverse cardiopulmonary effects of pollution. These are unique forms of risk mitigation, as they are based on behavioral changes at the level of the individual, rather than legislative or civic initiatives. Accordingly, they illuminate disparities – geographic, relating to availability and acquisition of resources, and otherwise – which complicate the applicability of these proposed interventions. Individual interventions can be classified by the location in which they are employed, including those employed in the home, those employed during travel, and those employed outdoors when exposed to ambient pollutants.

**Table 4:** Individual-level interventions to mitigate the effects of, and exposure to, pollutants.

Individual-level interventions	
Location of exposure	Interventions employed
Home [72], [81]	• Increased home ventilation • High-efficiency portable air filters (eg, HEPA filters) • Frequent replacement of high-efficiency air filters
In travel [72], [91]	• Keeping windows closed • Frequently recycle cabin air • Ensuring patent seal to vehicle • Prioritizing travel routes with lower pollutant burden
In response to outdoor pollutants [72]	• Avoiding outdoor activities during times of peak pollutant burden • Utilizing air pollution forecasting • Wearing a filtered mask (eg, N95, N99)

In response to the growing interest in the implications of pollution on cardiopulmonary health, an expert workshop, *Reducing the Cardiopulmonary Impact of Particulate Matter Air Pollution in High-Risk Populations*, identified several interventions, which could be employed at the individual level to mitigate long-term adverse cardiopulmonary events. Some of these interventions include wearing a filtered mask (eg, N95 or N99) in areas of elevated air pollutant levels, air pollution forecasting to avoid exposure during times of peak pollutant burden, increased ventilation at home, housing portable air purifiers, and the use of high-efficiency filters with frequent replacement [72].

The use of N95 respirators has been associated with reductions in systolic blood pressure (SBP) by 2.7 mmHg, lower mean arterial pressures (MAP), improved aortic hemodynamics (eg, aortic blood pressure and arterial stiffening), and reductions in exhaled nitrogen oxide and inflammatory cytokines (eg, IL-1α, IL-1β, and IL-6) [73], [74], [75], [76], [77], [78], [79]. N95 respirators have been shown to reduce PM_2.5_ exposure by a factor of 14 and hospitalizations by 22–39 % in settings of severe air pollution (eg, wildfires) [80].

High efficiency particulate air (HEPA) filters are air filters commonly employed in the home which can remove airborne particles with an efficacy of ≥99.97 % [81]. Studies have demonstrated approximately a 60 % reduction in home PM_2.5_ concentrations with the use of home HEPA filters [82], [83]. Meanwhile, non-HEPA home filters are associated with 46–52 % reductions in home PM_2.5_ concentrations [84], [85]. A 2021 systematic review and meta-analysis demonstrates an association of indoor HEPA filters with reduced SBP (−2.28 mmHg), diastolic blood pressure (−0.35 mmHg), pulse pressure (−0.86 mmHg), and C-reactive protein (−0.23 mg/dL) [86].

Air pollution forecasting is an increasingly important strategy used to mitigate exposure to pollutants during times of high pollution burden through the estimation of pollution burden for a geographic area during a given time frame [87]. Statistical forecasting models have demonstrated good predictive capability for PM, NO_2_, and O_3_ (R^2^ = 0.50–0.89) [88]. Deterministic approaches which provide a single forecast from a single model are being replaced by probabilistic ensemble predictions, which produce multiple salient forecasts for a location at a given time [89].

Probabilistic ensemble predictions have better predictive ability than deterministic approaches, with greater consistency demonstrated from consecutive forecasting compared to deterministic approaches [90]. Ensemble predictions, compared to single, high-resolution forecasts, estimate the probability of specific weather events in addition to the most probable weather event, situating itself as a more efficacious method of weather-risk management [90]. Forecasting strategies can be an invaluable tool, especially when used in conjunction with the EPA-derived Air Quality Index (AQI), a tool which associates an average daily PM_2.5_ level with an AQI score and risk of adverse health effects from PM_2.5_ exposure [91]. Forecasting strategies could predict days where PM_2.5_ exposures would be great enough to threaten harm to personal health – informing decisions to avoid prolonged outdoor activities. Endorsement of air pollution forecasting by the American Heart Association (AHA) derives from recommendations made by the EPA, based on data from the AQI [92].

Elevated indoor temperatures and humidity are associated with increased concentration of some pollutants, promoting passive air diffusion and concomitant dilution of indoor air pollutants. Further, ventilation should be strategically employed in home spaces posing the greatest risk for the accumulation of home pollutants. Common locations include the kitchen, which is predisposed to accumulate toxic levels of carbon monoxide, and small rooms; portable air purifiers are particularly salient in such spaces. On the other hand, high-efficiency filters reduce the risk for pollutant accumulation in all spaces within the home but require regular upkeep, maintenance, and replacement of filters for optimal efficacy [93].

While household ventilation reduces the risk of adverse effects from air pollutant accumulation, this action also poses the risk of introducing air pollutants originating outside of the home into home spaces [94]. Therefore, benefits of ventilation must be weighted with the potential for introduction of ambient pollutants when the preclusion of an incurrent cannot be guaranteed. For example, in housing with high levels of indoor pollution burden in areas of concurrent high ambient pollution burden, the risk of introducing ambient pollutants into the home must be considered in conjunction with the benefit of ventilating the home and reducing the in-home pollution burden. Additional interventions such as optimizing in-home combustion reactions (eg, cooking) through the addition of fans to cookstoves reduces emissions by up to 90% [95] – though, current data does not substantiate statistically-significant improvements in cardiorespiratory benefit [96].

Interventions employed during travel emphasize actions that limit the individual's exposure to ambient air pollutants as well as those that limit the generation and release of pollutants generated by vehicles. Proposed actions to minimize exposure to air pollutants while traveling include keeping windows closed when traveling through areas of high ambient air pollutant burden, frequently recycling cabin air, ensuring a patent seal on the vehicle to prevent the incurrent of air pollutants, and prioritizing travel routes which minimize the exposure to air pollutants during transportation.

The AHA recommends the avoidance of outdoor activities and the use of personal protection devices when the air quality index (AQI) is beyond hazardous (i.e., AQI >500) [91]. However, when avoidance is impossible (eg, necessity of travel), it is imperative to mitigate exposure. Closing windows and using cabin air filters reduces PM_2.5_ levels by 37 % and results in a congruent reduction in oxidative stress markers [97]. Multiple studies promote the use of high-efficiency cabin filters over manufacture-installed air filters, noting poor reduced filtering efficiency of ultrafine particles from manufacture-installed air filters when compared with high-efficiency cabin air filters (50 % vs 93 % reduction, respectively) [98], [99].

The AHA outlines notable limitations to current approaches to understanding and mitigating the risk of PM_2.5_-related disease. While the association between excessive PM_2.5_ exposure and CV morbidity and mortality has been well elaborated [28], current air pollution prevention guidelines do not address PM_2.5_ and associated preventive strategies [91]. Further, there is a paucity of randomized controlled clinical trials evaluating the efficacy of individual level mitigation strategies, and the potential roles of synergistic or additive effects of multiple air pollutants exposures has yet to be fully delineated [91].

### 2.6 Implications on planetary health

Planetary health refers to the health impacts of disruption to the earth's natural systems caused by humans [100]. Strategies to counter the health impacts of both air pollution and climate change have the potential to negatively impact the environment [13] resulting in further harm to population health (Table 5). For example, use of current air conditioning technology to protect against the health effects of extreme heat usually results in consumption of electrical energy generated by fossil fuel combustion with resultant greenhouse gas and air pollution production. The use of air filters, or any product, to mitigate generation of or exposure to pollutants, requires manufacturing. This would increase greenhouse gas emission secondary to industrial processes, which were responsible for 20 % of all greenhouse gas emissions in the US in 2020 [101].

**Table 5:** Planetary health implications of pollution mitigation strategies.

Planetary health implications of pollution mitigation strategies		
Mitigation strategy	Intention	Perturbation
Production of air filters [101]	Mitigate exposure to air pollutants	Increased production of greenhouse gases
Production of materials and goods for other mitigation strategies [105]	Mitigate exposure to various pollutants	Plastic used in packaging accumulates in various ecosystems and aquatic trophic levels ➔ human consumption

Multiple connections exist between climate change policy and fossil fuel combustion related to the industrial processes used to promote these initiatives, requiring that the implications of these interventions be considered from the perspective of how they could potentially exacerbate the effects they are designed to mitigate, even if these deleterious effects are only transient [102]. The World Induced Technical Change Hybrid (WITCH) integrated assessment model was developed to optimize climate change mitigation policies [103]. WITCH is an optimal growth hybrid model which aims to optimize a predetermined outcome by the input of control variables (eg, capital stocks, consumption of fossil fuels, energy technologies). As a model, it combines top-down and bottom-up models and accounts for economic interrelationships between the 12 regions it uses in its analysis [104]. WITCH data suggests optimized climate change mitigation policies are welfare maximizing policies which concomitantly account for air pollution benefits, with an estimated reduction of 1.62 million premature deaths [103].

Plastic is often employed in packaging of various goods, including those used in mitigation strategies, and the accumulation of plastics portends adverse consequences on human health [105]. Plastic accumulation is associated with perturbation of wildlife and the ecosystems they interact in, leeching of chemicals into soil – affecting soil composition and any food harvested from this land, as well as its secondary ingestion by humans – resulting from its primary ingestion by native animals which humans then consume [105].

Anthropogenic pollution has multiple ecological impacts; a meta-analysis demonstrated a reduction in species richness and diversity by up to 50 % in marine ecosystems with exposure to such pollution [106]. The adverse effects of anthropogenic pollutants on biodiversity threaten global biodiversity [107]. The human footprint implies that humans, directly and indirectly, influence ecosystem composition and interactions [108]. Human influences on ecosystem composition affects evolution and is likely to change with each interaction in the environment; this could refer to the production of pollutants or to the initiatives to promote their removal – each example will influence ecosystem composition and, in turn, influence humans' relationship with the environment [109]. Each intervention that impacts ecosystem function creates a potential threat on the viability of keystone species, those species which are the most important species for the maintenance of normal ecosystem function [110]. For example, the free-living pelagic larvae of the polar cod (*Boreogadus saida*), an arctic keystone species, are highly susceptible to the water-soluble fraction of crude oil introduced into their environment [111]. A 2016 study demonstrated a significant reduction in viability and fitness of the larvae of polar cod when exposed to aqueous hydrocarbons from oil (eg, oil introduced by human oil spills) [111]. Extinction of keystone species has been associated with secondary extinctions, which threaten human food sources, especially for those who rely on these species for nourishment [112].

## 3 Conclusion

Pollution is an important and complex CVD risk factor, posing both direct and indirect effects on human health. The admixture of pollutants burdening multiple areas and ecosystems portends considerable CVD risk, which must be addressed from multiple levels. Mitigating the harmful effects of pollution on CV health requires individuals and governmental bodies to implement sustainable strategies to prevent further pollution and address the current pollutant burden. Further studies are needed to better understand the health benefits of personal and population interventions to protect against pollutants. Studies are also required to evaluate the impact of these interventions in the broader context of their ecological impact and the influence they have on planetary health.

## CRediT authorship contribution statement

Earl Goldsborough III B.S.

• Conceptualization
• Resources
• Writing – original draft
• Writing – review & editing
• Visualization

Medha Gopal B.S.

• Resources
• Writing – review & editing
• Visualization

John William McEvoy M.B., B.CH. B.A.O. M.H.S. Ph.D.

• Resources
• Writing – review & editing

Roger S. Blumenthal M.D.

• Resources
• Investigation
• Writing – review & editing

Alan P Jacobsen M.B. B.CH. B.A.O.

• Conceptualization
• Resources
• Writing – original draft
• Writing – review & editing
• Visualization

All authors have contributed sufficiently to merit authorship.

## Declaration of competing interest

The authors declare that they have no known competing financial interests or personal relationships that could have appeared to influence the work reported in this paper.
